# Optimization of Cyanine Dye Stability and Analysis of FRET Interaction on DNA Microarrays

**DOI:** 10.3390/biology5040047

**Published:** 2016-11-30

**Authors:** Marcel von der Haar, Christopher Heuer, Martin Pähler, Kathrin von der Haar, Patrick Lindner, Thomas Scheper, Frank Stahl

**Affiliations:** Institute of Technical Chemistry, Leibniz University Hanover, Callinstr. 5, 30167 Hanover, Germany; christopher.heuer@gmx.net (C.H.); paehler@iftc.uni-hannover.de (M.P.); vonderhaar@iftc.uni-hannover.de (K.v.d.H.); lindner@iftc.uni-hannover.de (P.L.); scheper@iftc.uni-hannover.de (T.S.); stahl@iftc.uni-hannover.de (F.S.)

**Keywords:** microarray, DNA, scanning, photobleaching, fluorophore, cyanine dye, FRET, ROXS, bioinformatics, bioanalytics

## Abstract

The application of DNA microarrays for high throughput analysis of genetic regulation is often limited by the fluorophores used as markers. The implementation of multi-scan techniques is limited by the fluorophores’ susceptibility to photobleaching when exposed to the scanner laser light. This paper presents combined mechanical and chemical strategies which enhance the photostability of cyanine 3 and cyanine 5 as part of solid state DNA microarrays. These strategies are based on scanning the microarrays while the hybridized DNA is still in an aqueous solution with the presence of a reductive/oxidative system (ROXS). Furthermore, the experimental setup allows for the analysis and eventual normalization of Förster-resonance-energy-transfer (FRET) interaction of cyanine-3/cyanine-5 dye combinations on the microarray. These findings constitute a step towards standardization of microarray experiments and analysis and may help to increase the comparability of microarray experiment results between labs.

## 1. Introduction

DNA microarrays are a potent technology for high throughput gene regulation monitoring. Fluorescence-labeled complementary DNAs (cDNAs) are transcribed from mRNA which is acquired from different regulatory states of the chosen biological sample. These cDNAs are competitively hybridized on a modified glass slide. The differently labeled fluorophore cDNA-probes compete for binding spotted, immobilized DNA-targets. The ratio of the differently labeled, immobilized fluorophores on a spot therefore represents the relative abundance of RNA in the respective regulatory states. Technology based upon this principle has gained widespread use in molecular biology, genetics, and medicine [1,2], enabling high-throughput transcriptome analysis [3].

Nonetheless, DNA microarray technology is set back by a set of disruptive factors, limiting its application and potential exploitation. These technical, biochemical, and statistical biases are introduced in various steps of a DNA microarray experiment. Sequence-dependent bias is introduced by primer design [4], spot-geometry and homogeneity through choice of spotting technique, proximate humidity, and choice of buffer [5,6,7,8,9]. Further bias is introduced by choice of dyes, scanner settings, the presence/absence of ozone filters, the exposition to environmental light, etc. [10,11,12,13,14,15,16,17]. While significant results can still be acquired in spite of these bias sources, they still pose a substantial hindrance when it comes to lab to lab comparability and standardization [18].

This publication focuses on photonic and photochemical effects, such as photobleaching and energy transfer, that emerge while scanning the DNA microarrays. In previous works, photobleaching susceptibility of the almost omnipresent labeling agents cyanine-3 (Cy3) and cyanine-5 (Cy5) on DNA microarrays was investigated. Its effect on scanner data was successfully characterized and an empirical model was devised. The model efficiently normalizes the bias introduced by this effect with respect to the choice of dye, the previously carried out scans, and the scanner settings [19]. This study aims to minimize photobleaching of Cy3 and Cy5 using a reductive-oxidative, protective buffer (ROXS).

**Figure 1:**
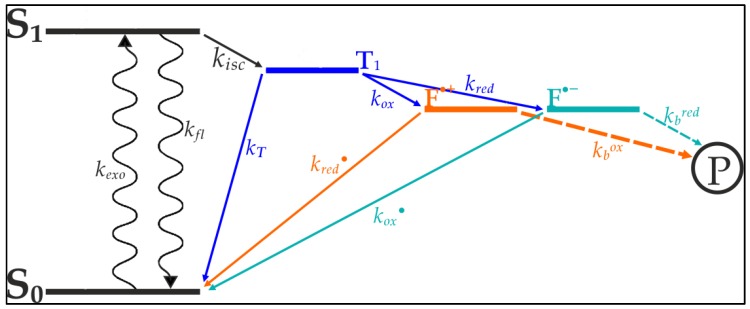
Schematic model of photoinduced electron excitation of organic fluorophores such as cyanine dyes. From its ground state (S_0_) the electron is excited to the first singlet state (S_1_). The sporadically forming triplet state (T_1_) is the point of origin for several transitions resulting in the formation of the photobleaching product (P). Methylviologen and ascorbic acid both rapidly deplete the T_1_, forming a radical cation (F^• +^ through methylviologen) or a radical anion (F^•−^ through ascorbic acid). These radical ions rapidly recover through reduction (ascorbic acid) or oxidation (methylviologen). The combination of an oxidizing agent and a reducing agent (ROXS) therefore minimizes photoinduced formation of P. Model, according to Vogelsang et al. [20].

Vogelsang et al. [20] were able to show that blinking and photo destruction of cyanine dyes could be significantly reduced through depopulation of reactive intermediate states of the cyanine’s exited electrons [20]. As seen in Figure 1, internal transitions of the cyanine’s exited electron lead to a triplet-state from which photo destruction originates. The depletion of this state minimizes the electrons availability for bleaching processes, thereby increasing the dye’s longevity and quantum yield. Based on the works of Widengren at al., Vogelsang et al. designed a buffer which contains an oxidizing agent and a reducing agent [21]. These agents are aimed at catalyzing the transition from the triplet-state towards the ground state (Figure 1).

Vogelsang et al. [20] and others [22] carried out their experiments in aqueous solution using a fluorescence microscope. This study aims to apply their findings in DNA microarray experiments, where the cyanine dye is bound to the DNA which itself is fixated on a modified glass slide and is scanned using a microarray slide scanner. This change in experimental design necessitated an adapted approach on array design and scanning technique. In order to allow for scanning in the presence of the protective ROXS-buffer, the arrays were partially modified by adding an improvised liquid chamber (see Section 2).

In addition, the possible occurrence of Förster-resonance-energy-transfer (FRET, also Fluorescence-Energy-Resonance-Transfer) in DNA microarrays and its implications on microarray analysis where examined. FRET between Cy3 and Cy5 molecules has already been described and is a common tool for oligonucleotide analysis [23]. Although commercial alternatives to the aforementioned cyanine dyes exist, Cy3 and Cy5 are still used ubiquitously, and the need for optimizing these dyes’ handling is compulsory.

**Figure 2:**
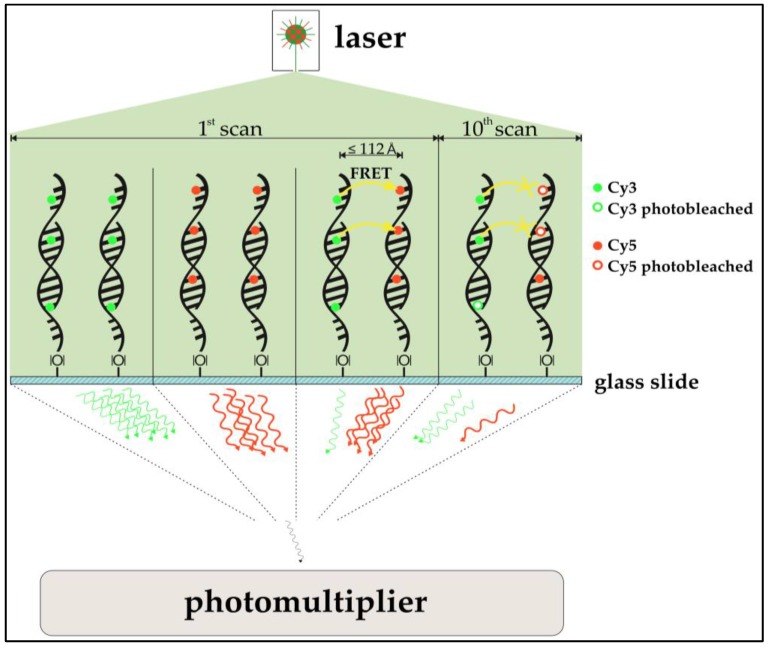
Schematic model of a hypothesized Förster-resonance-energy-transfer (FRET)-effect in cyanine-labeled two-dye DNA microarray scanning. Cyanine-labeled DNA in single-dye setups emits photons after excitation (upper left, upper mid left). In a two-dye setup at the first scan (upper mid right), Cy3 only partially emits photons after excitation. With Cy5 in it vicinity, Cy3 acts as a FRET donor, transferring the energy to the Cy5-acceptor, which itself emits a photon. This leads to a lower Cy3 signal and a higher Cy5 signal compared to Cy3 and Cy5 from single dye setups. After several scans, photobleaching should have decreased the amount of functional, photon-emitting cyanine molecules. While this would be observable in single dye setups (not shown), one does actually observe a different behavior in two-dye setups (upper left). The higher bleaching susceptibility of Cy5 decreased the chance of Cy3 acting as a FRET-donor, simultaneously increasing the amount of emitted photons from Cy3. While a strong decrease in Cy5-photon emission can be observed, the emission of Cy3 seems to have “increased”. This effect is called passive “de-quenching”. All emitted photons then enter the photomultiplier (PMT), where they are transformed into an exponentially enhanced electron signal.

The use of acceptor photobleaching (Cy5 being the acceptor) as a means to FRET validation is especially of interest for this study. In prior studies, the passive “de-quenching” of Cy3 by photo destruction of Cy5 resulted in an increase of donor (Cy3) photon emission, which was used to quantify FRET [24,25]. Among other findings, Rao et al. [26] qualitatively assessed if FRET is observable in DNA microarray two-dye experiments. To do so, a part of a spot containing Cy3- and Cy5-functionalized immobilized oligonucleotides was exposed to a confocal laser bleaching Cy5. Emissions of Cy3 and Cy5 were compared prior to and after the selective bleaching. In fact, the expected anti-proportional change in intensity for both dyes was observed, indicating that DNA microarray imaging of two-color experiments is biased by FRET. An investigation of the actual impact of FRET for multi-scan approaches is the subject of this study. Also, cross-over effects of FRET and ROXS protection are examined. A simplified model of the expected impact of the FRET-effect can be seen in Figure 2.

Other than to improve the awareness and understanding of underlying photochemical processes and their effect on microarray data, the results of these studies are aimed at the improvement of microarray bias minimization and the establishment of experiment reproducibility and lab-to-lab comparability.

## 2. Materials and Methods

**Oligo Preparation:** Single strand DNAs (ssDNA) of 50 nt length were purchased from Integrated DNA Technologies, Inc. (Munich, Germany). The sequences were optimized with regard to low stabilities of potential homodimers and hairpins. The 3′-end of the ssDNA was modified with an amino-modified C6 spacer. Ninety-six different sequences were used, corresponding to a set of 96 *Escherichia coli* genes. This set of genes was chosen because it provides a representative set of regulatory behaviors for heat-shock experiments. Also, the usage and analysis of these genes is well documented and routinely carried out in our workgroup. Information on these genes can be found in the Supplementary Materials (Table S1). The oligos were dissolved using Micro Spotting Solution Plus 2X from Arrayit Corporation (Sunnyvale, CA, USA) and nuclease free water to a final concentration of 100 mM (concentrations validated using a NanoDrop 2000 from Thermo Fisher Scientific Inc. (Waltham, MA, USA)). Solutions were stored at 4 °C.

**ROXS Buffer Preparation:** ROXS buffers were prepared freshly prior to each experiment. They were based on a 1× standard buffer from phosphate buffered saline (PBS) at pH 7.4, containing additional ascorbic acid (AA) and methylviologen dichloride hydrate (MV) at 100 mM each. Dilutions of this stock solution were prepared using 1× PBS. Consequently, if a buffer is described as, for example, 10 mM ROXS, it contains 10 mM AA and 10 mM MV in 1× PBS.

**Figure 3:**
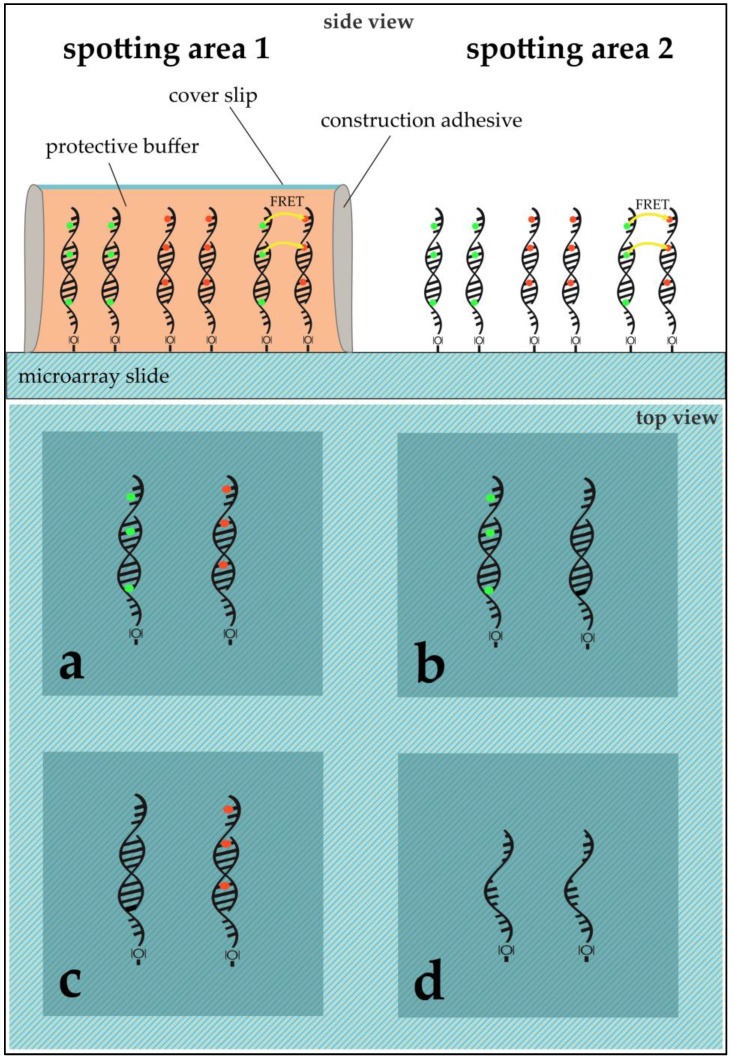
Microarray modified glass slide scheme for ROXS and FRET assessment. The slide shows two main spotting areas (**1**,**2**), each subdivided into four blocks (**a**–**d**). Each block was used to immobilize either 96 capture-oligos without replication or 24 capture-oligos with five replicates (six spots per gene). While (**a**) was used to hybridize Cy3- and Cy5-labeled cDNAs competitively; (**b**) for Cy3 vs. unlabeled cDNA; (**c**) for unlabeled vs. Cy5; and (**d**) was used as a negative control, where no hybridization took place. In the case of subsequent application of a protective cover slip, area **1** remained unprotected while area **2** was modified using a desired buffer and a cover slip (see **Application of PBS and/or ROXS-Buffer**).

**DNA Immobilization:** DNA sequences were immobilized on aldehyde modified glass slides (SuperAldehyde 2; Arrayit^®^ Corporation, Sunnyvale, CA, USA) using a non-contact-spotter (Nano Plotter™ NP2.1; GeSiM mbH, Großerkmannsdorf, Germany) with an applied voltage of 80 V. The selection of a contact-free printer allowed for higher homogeneity in spot geometry by avoiding pin-derived variance and providing humidity control in the spotting chamber (humidity at 60%). The general spotting layout can be found in Figure 3.

**RNA Treatment and On-Slide Hybridization:** RNA was purified and pooled from samples of two different treatments using Trizol reagent (Invitrogen, Karlsruhe, Germany) according to the manufacturer’s protocol. This method yielded an average of 30 µg total RNA from 10^6^ cells. In both cases, *E. coli* was cultivated until it reached the log-phase at 37 °C. While the 37 °C sample (Ec37) was obtained in this phase directly, the 50 °C sample (Ec50) *E. coli* was exposed to 50 °C for ten minutes before cell disruption and RNA purification. Fifty micromoles of purified DNA was transcribed into complementary DNA (cDNA) using a 1:1:1:1 unlabeled dNTP-mixture for unlabeled cDNA and a 1:1:1:0.25 unlabeled dNTP-mixture (with dCTP being the aforementioned 0.25) with the addition of 0.75 equivalents of Cy3- or Cy5-labeled dCTPs. In the case of labeling, Cy3 was always used for Ec37 while Ec50 was labelled with Cy5. The purified cDNAs were then competitively hybridized on the microarray slides. The hybridized microarray slides were put into cassettes, purchased from Arrayit Corporation, for microarray sample multiplexing. Sixteen microliters of the desired cDNA solution was pipetted into the wells (see Figure 3). The cassette’s wells were sealed using an adhesive strip to prevent dehydration and the arrays were hybridized at 100% humidity overnight. The slides were washed and dried through centrifugation.

**Application of PBS and/or ROXS-Buffer:** The spotting pattern allowed for two different treatments per slide. Possible treatments were: unprotected (bare slide, without any protection) or 1× PBS/10 mM ROXS/50 mM ROXS (40 µL of buffer were pipetted onto the slide, covered with a cover slip that was sealed using construction adhesive).

**Microarray Slide Scanning:** All scans were performed using the GenePix^®^ 4000B Microarray Scanner by Molecular Devices (Sunnyvale, CA, USA). All data was collected at a pixel size of 10 µm and a total resolution of 1891 × 2089 pixels. For unprotected areas the focus level remained by default at 0 µm. Areas protected by a cover slip were scanned at a focus level of 75 µm.

The usage of a different focus level for areas modified with a liquid film and a cover slip was imperative to maintain comparable imaging results. Each area was pre-scanned once to determine the scan-area and 10 additional scans of this area were performed at constant photomultiplier (PMT) voltages (635 nm-laser: 800 V, 532 nm-laser: 650 V) and 100% laser power. Data collection was carried out using GenePix^®^Pro 7.0 (Molecular Devices, Sunnyvale, CA, USA).

**Data Analysis:** After an initial quality control carried out by GenePix^®^Pro, all spots with any saturated pixels, as well as spots whose signal to noise ratio (SNR) was 3 or lower, were excluded from further analysis. The SNR is defined as follows:

(1) $$SNR=\;\frac{m_{Foreground}-m_{Background}}{s_{Background}}$$

where *m*: median; *s*: standard deviation.

Also, in accordance with Lyng et al.’s recommendations [15], spots with median local background subtracted intensities above 50,000 and below 1000 relative intensity units were excluded from further analysis to prevent saturation and/or noise bias. Although a correction for background is a general convention, the actual application varies. Background correction is carried out locally, within a sub-grid, with blank spots or control spots. Most of these approaches have different underlying assumptions on how the background intensity reflects an intensity bias over- or better underlying the feature intensity. Furthermore, Qin et al. [27] showed that while a background subtraction actually reduces the bias it increases data variability. The increase in variability is kept in check using the SNR threshold. Data was filtered and analyzed using MATLAB v7.12.0.635 (The MathWorks, Inc., Natick, MA, USA) and Visual Basic for Applications (Microsoft Corp., Redmond, WA, USA).

All derived statistical metadata in this study was calculated taking into account Gaussian error propagation. If not stated otherwise, all error indicators given in text and graphs always represent the respective value’s standard error of the mean (SEM) with a confidence level of 95.4%.

## 3. Results

### 3.1. Ninety-Six Gene Experiment

**Figure 4:**
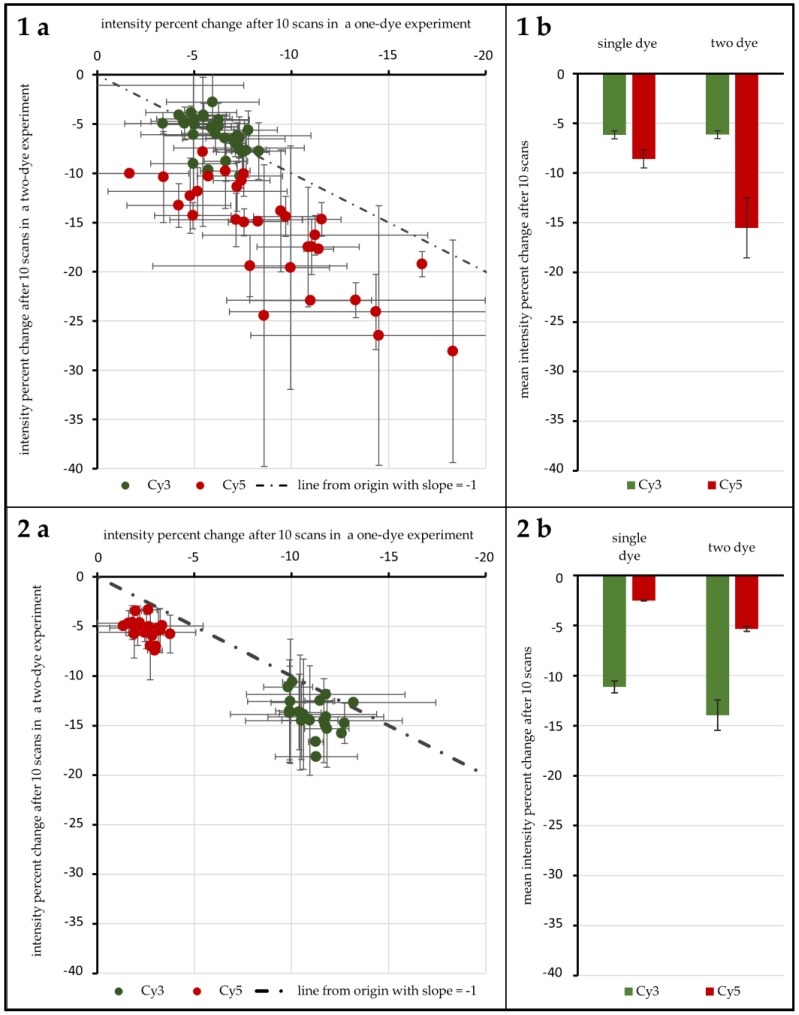
Results of the 96 gene experiment. The intensity percent change after 10 scans is compared for Cy3 and Cy5 depending on the presence/absence of their FRET partner. The percent change for one-dye setups is plotted against the same value derived from two dye experiments for unprotected spots (**1a**) and spots protected by 1 mM ROXS in 1× PBS (phosphate buffered saline) (**2a**). The dotted lines pass through the origin with a slope of −1. For each distribution the resulting means are given in (**1b**,**2b**). Error indicators are the respective standard errors (confidence: 95.4%) with Gaussian error propagation.

A first test with 96 genes was carried out to test the influence of ROXS as well as FRET and possible cross-over effects. As Figure 4(1a,b) shows, without any protective measures, spots hybridized with Cy3-labeled DNA lose 6.16% (±0.40%) in signal intensity on average after 10 scans when no Cy5-labeled DNA is present. Furthermore, 6.14% (±0.38%) are lost when Cy3-labeled DNA is hybridized competitively against Cy5-labeled DNA. T-Tests show that these two values cannot be considered different (α ≤ 0.1). For spots hybridized with Cy5-labeled DNA, on the other hand, a statistical difference is evident: hybridized against unlabeled DNA, Cy5-labeled DNA loses 8.61% (±0.90%) on average. When hybridized against Cy3-labeled DNA, the intensity decrease changes to 15.52% (±3.02%). These two means are significantly different for α ≤ 0.01. Statistical inquiries showed that the percentage intensity change evaluated here is independent of the initial intensity level of the spots under study, ruling out a possible intensity level bias (supporting data can be found in the Supplementary Materials, Tables S2 to S4). The use of protective measures (in this case 1 mM ROXS in 1× PBS) was evaluated in comparison (see Figure 4(2a,b). Here, Cy3 without Cy5 loses 11.14% (±0.60%), compared to 13.95% (±0.15%). This difference is as significant (α ≤ 0.01) as the change of single Cy5 with a loss of 2.51% (±0.05%) to Cy5 with Cy3 present, losing 5.34% (±0.24%).

### 3.2. Twenty-Four Gene Experiment

The 96 genes of the first experiment were spotted with one replica. The respective gene was analyzed only when sufficient data for all dye-combinations and treatments was available. The use of only one replica limited the amount of usable data (data from 61 genes could not be used because for at least one dye-combination/treatment only one spot met the quality criteria). In order to provide a more sufficient statistical basis to validate the 96 gene experiment and answer the remaining questions, a new experiment design was devised: from those 96 genes, 24 were selected that had the most stable and homogenous spots, also providing a signal variety concerning overall intensity level and Cy3/Cy5-intensity-ratios. The corresponding 24 oligos where spotted with five replications, providing a solid basis for statistical analysis.

In addition to the evaluation of unprotected areas, several protective measures were compared: liquid chamber with 1× PBS, liquid chamber with 10 mM ROXS, and liquid chamber with 50 mM ROXS. With respect to array-to-array variability, each slide held one unprotected area and one protected area, to allow for array-to-array comparisons via normalization of the unprotected areas, resulting in the following pattern: Array1: unprotected vs. 1× PBS, Array2: unprotected vs. 10 mM ROXS in PBS, Array3: unprotected vs. 50 mM ROXS in PBS.

**Figure 5:**
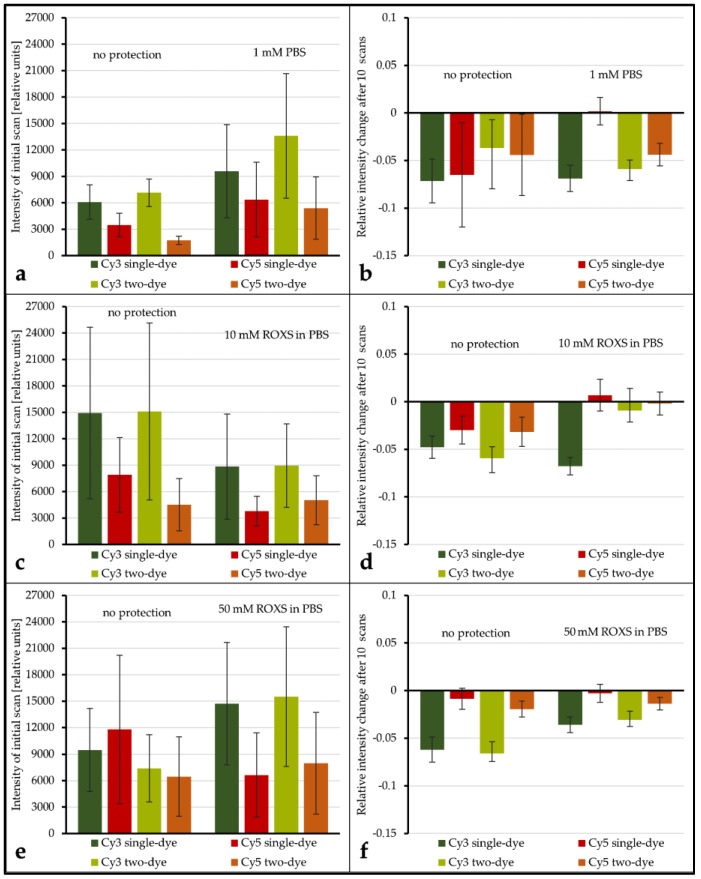
Examination of the influence of protective measures on the mean spot intensity level and the spot intensity percent change after 10 scans. Intensity levels are derived from Array1: unprotected vs. PBS (**a**), Array2: unprotected vs. 10 mM ROXS (**c**), and Array3: unprotected vs. 50 mM ROXS (**e**). Spot intensity percent changes after 10 scans are derived from Array1: unprotected vs. 1x PBS (**b**), Array2: unprotected vs. 10 mM ROXS (**d**), and Array3: unprotected vs. 50 mM ROXS (**f**). Intensity information derived from hybridizations of Cy3/Cy5 vs. unlabeled cDNA is tagged “single-dye” while intensity information derived from hybridization of Cy3-labeled cDNA with Cy5-labeled cDNA is tagged “as two-dye”. Error indicators are the respective standard errors of the mean (confidence: 95.4%) with Gaussian error propagation.

In a first evaluation of the results it could be confirmed that the three arrays’ unprotected spots are statistically comparable with respect to intensity level, percentage intensity change, and overall spot intensity standard deviation (Table 1 and Table 2, Supplementary Materials ANOVAs, Tables S2 to S4). Even if the application of a liquid chamber and/or ROXS reduces photobleaching, it is important to investigate if this modification is beneficial for gathering microarray data in general. To shed light on this subject, the spot intensity level as well as the spot’s initial intensity deviation of unprotected spots were compared to their protected counterparts (intensity level: see Figure 5a,c,e, intensity deviation: see Supplementary Materials, Figure S1). The average intensity level seems to decrease for protected spots in some cases (Figure 5c,e) and seems to increase in others (Figure 5a,e). However, because of the overall broad distribution of intensity levels within each group, these decreases and increases are never significant, even for α = 0.1. Comparing the intensity deviations of protected and unprotected spots by Analysis of Variance (ANOVA) showed no significant change.

The evaluation of spot intensity percent change for unprotected vs. PBS shows that, for all but one combination of dyes, no significant change in intensity percent change is observable (for α ≤ 0.1). Only for Cy5 vs. unlabeled the application of the liquid chamber significantly (α = 0.05) decreases the average intensity percent change from −6.51% (±5.47%) to 0.18% (±1.45%). Conversely, for unprotected vs. 10 mM ROXS, all dye combinations see a significant (α ≤ 0.05) elevation of percent change levels. Most of them are even significant for α ≤ 0.01. While the application of 50 mM ROXS leads to significant reduction of intensity loss for Cy3-labeled DNAs (α ≤ 0.01), this cannot be concluded for their Cy5-labeled counterparts. It should be noted that percent changes for Cy5 on this array were lower in general, compared to the other arrays.

While there are singular significant differences when comparing the intensity level and intensity percent change of a labeled DNA of a single dye spot with its two-dye counterpart, the overall results show that no significant differences (α ≤ 0.1) exist in this 24 gene experiment. Similar to the ROXS results, the statistical analysis of the intensity level is limited by the overall broad dynamic range of intensity level within each group.

An evaluation of the impact of FRET on actual log-ratios was carried out to investigate the impact of FRET and/or protective measures on actual microarray data analysis. Log ratios (with base 2) of single labeled Cy3 and single labeled Cy5 spots were compared to log ratios derived from their respective Cy3. vs. Cy5 labeling counterparts.

(2) log2ratio(Cy5/Cy3)= log2(Intensity Cy5Intensity Cy3)

**Figure 6:**
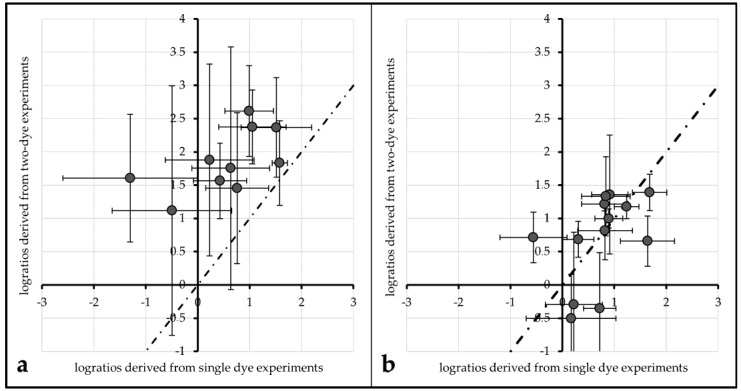
Comparison of log_2_-ratios derived from single-dye spots (635 nm intensity from Cy3 vs. unlabeled, 532 nm intensity from unlabeled vs. Cy5) plotted against log_2_-ratios derived from two-dye spots for unprotected spots of Array2 (**a**) and spots protected by 10 mM ROXS in PBS (**b**). The dotted line passes the origin with a slope of 1.

For unprotected spots of Array2, the average log-ratio derived from Cy3-single intensity divided by Cy5-single intensity was 0.54 (±0.45), while the average log-ratio from two-dye spots was 1.86 (±0.66). These two values differ significantly (α ≤ 0.01). Plotted against each other, all data points lie above a line from the origin with a slope of 1 (see Figure 6a).

This tendency can also be observed for the same comparisons made with 1× PBS protected spots of Array1 (single dye log ratio: 0.75 (±0.28), two-dye log ratio: 1.53 (±0.23)) and unprotected spots of Array2 (single dye log ratio: 0.72 (±0.35), two-dye log ratio: 1.64 (±0.37)). For both treatments, single dye log ratios are significantly different (α ≤ 0.01) from two-dye log ratios.

Comparing the same values for ROXS-treated spots of Array2 gives different results: the mean log ratios of single dyes (0.99 (±0.23)) are not significantly different from those of two-dye spots (0.91 (±0.26)) for α ≤ 0.1 (graphical representation: Figure 6b).

## 4. Discussion

The importance of reliable bias normalization and quality control is well recognized in the microarray field. Next to biological and biochemical sources, bias originates from photochemical processes and depends on the choice of labeling agent as well as the selected imaging procedure and environment. In earlier works, it was shown that the ubiquitous application of cyanine dye labeling causes significant bias due to the dyes’ disparate susceptibility to photobleaching and possible FRET interaction [19,20,26,28,29].

These findings are confirmed in this study, as photobleaching and FRET result in significantly different data: as shown in Figure 4(1a,b), without protective measures, photobleaching occurs similar to previous findings of von der Haar et al. [19] with intensity decreases for Cy3 and to a higher degree for Cy5. Interestingly, the 96 gene experiment shows that these photobleaching percentages nearly switch when applying a wet chamber with ROXS. A comparably higher decrease for Cy3 intensity and a lower intensity decrease for Cy5 is observed. Both changes are statistically significant. These findings confirm those of Vogelsang et al. [20], especially the reduction of Cy5 intensity loss. The increase of Cy3-intensity loss is significantly stronger for Cy3-labeled DNA hybridized against Cy5-labeled DNA. It can be primarily traced back to a hypothesized FRET effect. This passive “de-quenching” effect, which has similarly been described by Rao et al. [26], was observable for a variety of genes, independent of the initial spot intensity (Supplementary Materials, SF1). In one case (Figure 4(1)), the higher photobleaching susceptibility of Cy5 most probably decreased the chance of excited Cy3 electrons to pass their energy over to nearby Cy5 molecules through FRET. This would result in a higher Cy3 emission that partially negates the intensity-decreasing effect of its own Cy3-photobleaching. In the presence of ROXS (Figure 4(2)), however, Cy5 would especially be protected from photo-destruction (see Rao et al. [26]). This would keep the rate of FRET between the two dyes stable, resulting in a visibly bigger and statistically significant intensity decrease of Cy3, which is not masked by “de-quenching” effects, as seen in Figure 4. Therefore, it is assumed that the de-quenching was observable not because of the selective bleaching of one cyanine dye.

Much of the reasoning applied above is based on the hypothesis that FRET happens to an observable degree in microarray experiments. In order to confirm FRET and quantify the effect, the experiment design was adapted so that for each bleaching condition there were spots with only Cy3-labeled DNA hybridized against unlabeled DNA, only Cy5-labeled vs. unlabeled, as well as Cy3-labeled vs. Cy5-labeled. In Figure 4(1a), the percentage of intensity change of the single dye spots after 10 scans is plotted against the same value for two-dye-spots for both cyanine dyes, respectively. If FRET does not occur to an observable degree, the absence/presence of a second dye would have no influence on the intensity change of the first. Data points for both dyes should then be scattered normally distributed around a spot/gene-specific point on a line from the origin with a slope of −1; however, this is only the case for Cy3. It was found that unprotected Cy3-labeled DNA did not show a significantly different intensity decrease depending on the presence/absence of Cy5-labeled DNA. It is hypothesized that the overall low observable bleaching of this group’s spots obscures possible FRET effects. For unprotected Cy5-labeled DNA, however, the intensity decrease is significantly higher when Cy3-labeled DNA is present. A possible explanation is the higher rate of excitation of Cy5 due to FRET which subsequently leads to more chances of Cy5-photobleaching. For ROXS-protected spots, we see significantly higher bleaching for two-dye spots of both Cy3 and Cy5. While the explanation for a visible hypothesized FRET effect on Cy3 has been stated above, the question of why a hypothesized FRET effect is observable for Cy5 remains. Compared to unprotected Cy3 DNA, it is expected that the comparably overall low level of protected Cy5 intensity decrease results in a similar insignificant observable difference of intensity decrease of single-dye and two-dye spots. The fact that a significantly higher decrease is still observable for Cy5 of two-dye spots can be explained by referring to the cDNA labeling and scanner settings: although Cy5-molecules are only different in structure by one conjugated C-C double bond, Cy5’s direct labeling efficiency is significantly lower compared to the one of Cy3. This source of possible bias is mostly addressed by adjusting/increasing the photomultiplier voltage of the 635 nm laser to lift the intensity level of Cy5-signals to the one of Cy3-signals. As the process of photo multiplication exponentially enhances the photon signal, the comparably higher voltage applied to Cy5-emitted photons might lead to a non-linear enhancement of intensity resolution. Consequently, two differently intense Cy5 photon signals might result in a larger observed intensity difference than that of two equally different Cy3 signals. While a stronger bleaching of Cy5 is to be expected, the data of the 24 gene experiment does not support this hypothesis. In all cases, Cy3 loses a higher fraction of its intensity after ten scans. Concerning the setups with ROXS present, this protective buffer has a stronger preserving effect on Cy5 than on Cy3, as described in the literature [20]. This could result in comparably higher observable relative Cy3 intensity loss. The results of unprotected spots of the 24 gene experiment should show a higher relative intensity change for Cy5, as they do in the 96 gene experiment (Figure 5b). Why this is not the case remains unclear.

Overall, this experiment statistically supports the hypothesis that FRET is an observable effect in DNA microarrays. Statistical inquiries showed that the percentage intensity change evaluated here is independent of the initial intensity level of the spot under study, ruling out a possible intensity level bias, as first evaluations showed that the overall intensity level is decreased by applying a liquid chamber onto the array (additional data can be found in the Supplementary Materials, Tables S2 to S4).

After the evaluation of this experiment, questions remained: If the increase of observable Cy3-intensity loss is explained by FRET effects, why does it also occur, to a lesser extent, in single-dye setups? To what extent is the observed reduction of photobleaching caused by ROXS? Are the dyes merely protected due to the application of the liquid chamber itself? This would indicate that the bleaching is mostly caused by environmental ozone that is now efficiently blocked. A first evaluation of the 24 gene experiment showed that the data derived from unprotected spots of all three tested arrays are statistically comparable with respect to intensity level, percentage intensity change, and overall spot intensity standard deviation. This, in theory, allows for further examination and comparison of spots from different arrays; therefore, additional array-to-array normalization was not carried out, which might also introduce bias obfuscating FRET and/or ROXS effects.

The application of a liquid chamber did not influence the overall intensity levels or the magnitude of the dynamic signal intensity range for both dyes. While this is a desirable outcome regarding the intensity level, an effect on the dynamic range would have been a mixed blessing: increasing the dynamic range benefits the resolution and therefore the distinguishability on the one hand, but increases the need for problematic multi-scan applications to cover this broadened range on the other. A decrease would have the contrary effect, sacrificing resolution for more convenient scanning.

In order to ensure that the observed photobleaching protection is due to the ROXS buffer components, the 24 gene experiment’s setup included spot protected by a liquid chamber filled with PBS buffer without ROXS. All comparison of intensity percent change made for unprotected vs. PBS protected spots showed no significant reduction of intensity loss through application of a liquid chamber with PBS buffer. The only exception was the single Cy5 DNA, though for a less significant threshold of α = 0.05. Comparing unprotected spots with spots protected by a liquid chamber filled with 10 mM ROXS in PBS, however, displayed a highly significant reduction of intensity loss for all compared configurations. These findings strongly indicate that the presence of 10 mM ROXS is actually responsible for the changes observed in the 96 gene experiment. The additional test of unprotected vs. 50 mM ROXS did not yield conclusive results as a significant reduction of intensity loss was only observed for Cy3 and not for Cy5, though overall low intensity of Array3’s spots might have affected the statistical power of these specific results. On the other hand, a 10 mM solution of ROXS is closer to the 1 mM formula used by Vogelsang et al. [20].

In contradiction with the 96 gene experiment, the application of ROXS in the 24 gene experiment also significantly reduced bleaching of Cy3-labeled DNA. Whether this change in observed behavior was due to the changed ROXS concentration or merely resulted from the absence of bias due to the better statistical power of the 96 gene experiment’s design cannot be ascertained at this point. All in all, these results show that the application of a liquid chamber filled with a 1 mM or 10 mM ROXS solution provides a practical solution for significant reduction of cyanine dye photobleaching caused by DNA microarray scanning.

Regarding FRET, the same parameters used to evaluate this effect in the 96 gene experiment do not yield the expected results in the 24 gene experiment. Only 2 out of 12 comparisons showed a significantly different intensity percent change of a dye depending on the presence/absence of its cyanine counterpart. This might mislead the observer to the conclusion that the FRET influence observed in the 96 gene experiment is a bias which disappeared due to the better statistical power. A closer investigation of the effect FRET has on the results of a typical analysis carried out with the 24 gene experiment’s data gives a different picture: log_2_-ratios derived from single-dye data in comparison with two-dye data were plotted against each other (Figure 6). For data derived from unprotected and PBS protected spots, the data points do not seem to be normally distributed around a line to the origin with a slope of 1. Normal distribution around this slope would be the expected result if the spots were not affected by FRET. This impression is statistically proofed as the means of single-dye spots of unprotected/PBS-protected spots are significantly different from those of two-dye spots of the same treatment. Carrying out the same comparison for ROXS-protected spots of Array2 gives a different result: the mean log_2_-ratios of single-dye spots are not significantly different from their two-dye equivalents. These observations support the theory that FRET is not only occurring in two-dye microarrays, it is significantly biasing the results of these experiments. Furthermore, the FRET-induced bias seems to be normalized by applying the ROXS-protection, as no significant difference can be observed in this case. Therefore, the application of a ROXS-filled liquid chamber seems not only to be beneficial in terms of photobleaching minimization but also poses a valid strategy in order to normalize FRET-dependent bias in two-dye experiments.

In order to allow for this novel technology to be used in daily experiments, several investigations and optimizations remain. Is the remaining variability of log_2_-ratios caused by the difference of the treatments/dye usages or by systematic/technical variance? If the application of ROXS does actually compensate the bias introduced by FRET, as implicated by the 24 gene experiment’s results, further investigations are necessary. Does ROXS minimize the occurrence of FRET by minimizing the availability of the specific excited electron state from which FRET is initiated in Cy3, much like with the photobleaching initiating states? Or, is FRET still occurring but the presence of ROXS implements another compensating effect? FRET is described as induced oscillation of two excited singlet-state electrons, while photodestruction originates from a triplet-state. Further replication of the experiments carried out in this study is needed to allow for quantification of FRET influences, leading to predictive models. Additionally, further tests are necessary to determine the optimal ROXS and buffer concentrations for DNA experiments. Future research should also broaden the application of this approach to protein and cellular microarrays. This necessitates the examination of ROXS’s effect on protein-stability and the compounds’ biocompatibility.

## 5. Conclusions

Based on the findings of Vogelsang et al. [20], Rao et al. [26], and our own previous research [19], a novel strategy for the minimization of photobleaching in cyanine-labeling-based DNA microarray experiments was successfully implemented. The modification of DNA microarray slides with thin liquid chambers filled with a buffer containing ROXS provided a valid protection of cyanine dyes against photo destruction occurring in the scanning process. Furthermore, it was shown that while FRET does not only occur in DNA microarray experiments, it does significantly bias the results of two-dye microarray derived data. This bias can successfully be normalized by applying the same ROXS-buffer-filled liquid chamber to the microarray slide. With necessary further optimization of this technology, the photonic limitations of cyanine-based microarray scanning can be overcome. This does not only improve the reproducibility of these experiments, it allows for successful implementation of multi-scan approaches with all the resulting possibilities.

## Figures and Tables

**Table 1:** Results and statistical metadata regarding the mean initial spot intensity (I0) of the 24 gene experiment.

Array	Dye Combination	Mean I0	Std. Error α = 95.4%	Mean I0	Std. Error α = 95.4%	*p*-Value
1		unprotected		PBS		
	Cy3 single dye	6.08 × 10^3^	1.96 × 10^3^	9.58 × 10^3^	5.30 × 10^3^	2.28 × 10^−1^
	Cy5 single dye	3.47 × 10^3^	1.34 × 10^3^	6.37 × 10^3^	4.22 × 10^3^	2.03 × 10^−1^
	Cy3 two dye	7.14 × 10^3^	1.57 × 10^3^	1.36 × 10^4^	7.07 × 10^3^	8.79 × 10^−2^
	Cy5 two dye	1.74 × 10^3^	4.64 × 10^2^	5.39 × 10^3^	3.54 x 10^3^	5.26 × 10^−2^
2		unprotected		10 mM ROXS in PBS		
	Cy3 single dye	1.49 × 10^4^	9.73 × 10^3^	8.83 × 10^3^	5.98 × 10^3^	3.23 × 10^−1^
	Cy5 single dye	7.90 × 10^3^	4.24 × 10^3^	3.77 × 10^3^	1.68 × 10^3^	1.02 × 10^−1^
	Cy3 two dye	1.51 × 10^4^	1.00 × 10^4^	8.96 × 10^3^	4.74 × 10^3^	3.08 × 10^−1^
	Cy5 two dye	4.51 × 10^3^	2.98 × 10^3^	5.02 × 10^3^	2.77 × 10^3^	8.12 × 10^−1^
3		unprotected		50 mM ROXS in PBS		
	Cy3 single dye	9.48 × 10^3^	4.71 × 10^3^	1.47 × 10^4^	6.95 × 10^3^	2.58 × 10^−1^
	Cy5 single dye	1.18 × 10^4^	8.40 × 10^3^	6.63 × 10^3^	4.75 × 10^3^	3.73 × 10^−1^
	Cy3 two dye	7.39 × 10^3^	3.80 × 10^3^	1.55 × 10^4^	7.91 × 10^3^	9.03 × 10^−2^
	Cy5 two dye	6.46 × 10^3^	4.49 × 10^3^	7.97 × 10^3^	5.76 × 10^3^	7.08 × 10^−1^

**Table 2:** Results and statistical metadata regarding the mean spot intensity percent change after 10 scans (relative ∆ I0) of the 24 gene experiment.

Array	Dye Combination	Relative ∆ I0	Std. Error α = 95.4%	Relative ∆ I0	Std. Error α = 95.4%	*p*-Value
1		unprotected		PBS		
	Cy3 single dye	−7.15 × 10^−2^	2.31 × 10^−2^	−6.88 × 10^−2^	1.37 × 10^−2^	8.45 × 10^−1^
	Cy5 single dye	−6.51 × 10^−2^	5.47 × 10^−2^	1.81 × 10^−2^	1.45 × 10^−2^	2.67 × 10^−2^
	Cy3 two dye	−3.68 × 10^−2^	2.97 × 10^−2^	−5.91 × 10^−2^	9.39 × 10^−3^	1.67 × 10^−1^
	Cy5 two dye	−4.41 × 10^−2^	4.28 × 10^−2^	−4.38 × 10^−2^	1.19 × 10^−2^	9.88 × 10^−1^
2		unprotected		10 mM ROXS in PBS		
	Cy3 single dye	−4.77 × 10^−2^	1.17 × 10^−2^	−6.77 × 10^−2^	9.30 × 10^−3^	1.68 × 10^−2^
	Cy5 single dye	−2.98 × 10^−2^	1.48 × 10^−2^	6.88 × 10^−3^	1.67 × 10^−2^	3.80 × 10^−3^
	Cy3 two dye	−5.94 × 10^−2^	1.19 × 10^−2^	−9.42 × 10^−3^	2.36 × 10^−2^	1.01 × 10^−3^
	Cy5 two dye	−3.16 × 10^−2^	1.54 × 10^−2^	−1.93 × 10^−3^	1.19 × 10^−2^	7.35 × 10^−3^
3		unprotected		50 mM ROXS in PBS		
	Cy3 single dye	−6.19 × 10^−2^	1.30 × 10^−2^	−3.60 × 10^−2^	8.31 × 10^−3^	8.59 × 10^−3^
	Cy5 single dye	−8.72 × 10^−3^	1.12 × 10^−2^	−2.84 × 10^−3^	9.58 × 10^−3^	4.89 × 10^−1^
	Cy3 two dye	−6.58 × 10^−2^	1.21 × 10^−2^	−3.10 × 10^−2^	9.46 × 10^−3^	6.78 × 10^−4^
	Cy5 two dye	−1.95 × 10^−2^	8.40 × 10^−3^	−1.38 × 10^−2^	6.60 × 10^−3^	3.64 × 10^−1^

## Supplementary Materials

The following are available online at www.mdpi.com/2079-7737/5/4/47/s1, Figure S1: Influence of spot intensity level on spot intensity percent change after 10 scans. (a) unprotected single dye spots; (b) unprotected two dye spots; (c) 1 mM ROXS in 1 mM PBS protected single dye spots; (d) 1 mM ROXS in 1 mM PBS protected two dye spots. Error indicators are simple standard deviations; Table S1: Sets of Genes for which oligos where designed as used in the 96 gene, two-array experiment and the 24 gene, three-array experiment; Table S1: Influence of presence absence of protective measures on overall spot intensity deviations for Array1 (unprotected vs. 1 mM PBS). ***SS***: Sum of Squares, ***df***: degrees of freedom, ***MS***: Mean of Square Sums, ***F***: F-value, ***p***: p-value corresponding to *F*, ***F_crit_***: critical *F* corresponding to chosen confidence interval (*α* = 0.05); Table S2: Influence of presence absence of protective measures on overall spot intensity deviations for Array2 (unprotected vs. 10 mM ROXS in 1 mM PBS). ***SS***: Sum of Squares, ***df***: degrees of freedom, ***MS***: Mean of Square Sums, ***F***: F-value, ***p***: p-value corresponding to *F*, ***F_crit_***: critical *F* corresponding to chosen confidence interval (*α* = 0.05); Table S3: Influence of presence absence of protective measures on overall spot intensity deviations for Array2 (unprotected vs. 10 mM ROXS in 1 mM PBS). ***SS***: Sum of Squares, ***df***: degrees of freedom, ***MS***: Mean of Square Sums, ***F***: F-value, ***p***: p-value corresponding to *F*, ***F_crit_***: critical *F* corresponding to chosen confidence interval (*α* = 0.05); Table S4: Influence of presence absence of protective measures on overall spot intensity deviations for Array3 (unprotected vs. 50 mM ROXS in 1 mM PBS).

## References

[1] Spielbauer B., Stahl F. (2005). Impact of Microarray Technology in Nutrition and Food Research. Mol. Nutr. Food Res..

[2] Allison D.B., Cui X., Page G.P., Sabripour M. (2006). Microarray Data Analysis: From Disarray to Consolidation and Consensus. Nat. Rev. Genet..

[3] Ehrenreich A. (2006). DNA Microarray Technology for the Microbiologist: An Overview. Appl. Microbiol. Biotechnol..

[4] Kretschy N., Somoza M.M. (2014). Comparison of the Sequence-Dependent Fluorescence of the Cyanine Dyes Cy3, Cy5, Dylight Dy547 and Dylight Dy647 on Single-Stranded DNA. PLoS ONE.

[5] Mary-Huard T., Daudin J.J., Robin S., Bitton F., Cabannes E., Hilson P. (2004). Spotting Effect in Microarray Experiments. BMC Bioinform..

[6] Dawson E.D., Reppert A.E., Rowlen K.L., Kuck L.R. (2005). Spotting Optimization for Oligo Microarrays on Aldehyde-Glass. Anal. Biochem..

[7] Sobek J., Aquino C., Weigel W., Schlapbach R. (2013). Drop Drying on Surfaces Determines Chemical Reactivity—The Specific Case of Immobilization of Oligonucleotides on Microarrays. BMC Biophys..

[8] Rao A.N., Grainger D.W. (2014). Biophysical Properties of Nucleic Acids at Surfaces Relevant to Microarray Performance. Biomater. Sci..

[9] Jang H., Cho M., Kim H., Kim C., Park H. (2009). Quality Control Probes for Spot-Uniformity and Quantitative Analysis of Oligonucleotide Array. J. Microbiol. Biotechnol..

[10] Khondoker M.R., Glasbey C.A., Worton B.J. (2006). Statistical Estimation of Gene Expression Using Multiple Laser Scans of Microarrays. Bioinformatics.

[11] Satterfield M.B., Lippa K., Lu Z.Q., Salit M.L. (2008). Microarray Scanner Performance over a Five-Week Period as Measured with Cy5 and Cy3 Serial Dilution Slides. J. Res. Natl. Inst. Stand. Technol..

[12] Ambroise J., Bearzatto B., Robert A., Macq B., Gala J.L. (2012). Combining Multiple Laser Scans of Spotted Microarrays by Means of a Two-Way Anova Model. Stat. Appl. Genet. Mol. Biol..

[13] Vora G.J., Meador C.E., Anderson G.P., Taitt C.R. (2008). Comparison of Detection and Signal Amplification Methods for DNA Microarrays. Mol. Cell. Probes.

[14] Shi L., Tong W., Su Z., Han T., Han J., Puri R.K., Fang H., Frueh F.W., Goodsaid F.M., Guo L. (2005). Microarray Scanner Calibration Curves: Characteristics and Implications. BMC Bioinform..

[15] Lyng H., Badiee A., Svendsrud D.H., Hovig E., Myklebost O., Stokke T. (2004). Profound Influence of Microarray Scanner Characteristics on Gene Expression Ratios: Analysis and Procedure for Correction. BMC Genom..

[16] Dar M., Giesler T., Richardson R., Cai C., Cooper M., Lavasani S., Kille P., Voet T., Vermeesch J. (2008). Development of a Novel Ozone- and Photo-Stable Hyper5 Red Fluorescent Dye for Array Cgh and Microarray Gene Expression Analysis with Consistent Performance Irrespective of Environmental Conditions. BMC Biotechnol..

[17] Kuang C., Luo D., Liu X., Wang G. (2013). Study on Factors Enhancing Photobleaching Effect of Fluorescent Dye. Measurement.

[18] Drăghici S. (2011). Statistics and Data Analysis for Microarrays Using R and Bioconductor.

[19] Von der Haar M., Preuß J.A., von der Haar K., Lindner P., Scheper T., Stahl F. (2015). The Impact of Photobleaching on Microarray Analysis. Biology.

[20] Vogelsang J., Kasper R., Steinhauer C., Person B., Heilemann M., Sauer M., Tinnefeld P. (2008). A Reducing and Oxidizing System Minimizes Photobleaching and Blinking of Fluorescent Dyes. Angew. Chem. Int. Ed. Engl..

[21] Widengren J., Chmyrov A., Eggeling C., Lofdahl P.A., Seidel C.A.M. (2007). Strategies to Improve Photostabilities in Ultrasensitive Fluorescence Spectroscopy. J. Phys. Chem. A.

[22] Van der Velde J.H., Oelerich J., Huang J., Smit J.H., Hiermaier M., Ploetz E., Herrmann A., Roelfes G., Cordes T. (2014). The Power of Two: Covalent Coupling of Photostabilizers for Fluorescence Applications. J. Phys. Chem. Lett..

[23] Sapsford K.E., Berti L., Medintz I.L. (2006). Materials for Fluorescence Resonance Energy Transfer Analysis: Beyond Traditional Donor-Acceptor Combinations. Angew. Chem. Int. Ed..

[24] Sabanayagam C.R., Eid J.S., Meller A. (2005). Using fluorescence resonance energy transfer to measure distances along individual dna molecules: Corrections due to nonideal transfer. J. Chem. Phys..

[25] Dinant C., van Royen M.E., Vermeulen W., Houtsmuller A.B. (2008). Fluorescence Resonance Energy Transfer of Gfp and Yfp by Spectral Imaging and Quantitative Acceptor Photobleaching. J. Microsc..

[26] Rao A.N., Rodesch C.K., Grainger D.W. (2012). Real-Time Fluorescent Image Analysis of DNA Spot Hybridization Kinetics to Assess Microarray Spot Heterogeneity. Anal. Chem..

[27] Qin L.X., Kerr K.F., Contributing Members of the Toxicogenomics Research Consortium (2004). Empirical Evaluation of Data Transformations and Ranking Statistics for Microarray Analysis. Nucleic Acids Res..

[28] Rao A.N., Rodesch C.K., Grainger D.W. (2013). Supporting Information of Real-Time Fluorescent Image Analysis of DNA Spot Hybridization Kinetics to Assess Microarray Spot Heterogeneity (Vol 84, Pg 9379, 2012). Anal. Chem..

[29] Rao A.N., Vandencasteele N., Gamble L.J., Grainger D.W. (2012). High-Resolution Epifluorescence and Time-of-Flight Secondary Ion Mass Spectrometry Chemical Imaging Comparisons of Single DNA Microarray Spots. Anal. Chem..

