# Supplementary material for: Optimization of Cyanine Dye Stability and Analysis of FRET Interaction on DNA Microarrays
Source: Biology (Basel). 2016 Nov 30;5(4):47. doi: 10.3390/biology5040047 (PMC5192427; doi:10.3390/biology5040047)
Supplement: Supplementary file 1 [file biology-05-00047-s001.docx]

Supplementary Materials: Optimization of Cyanine Dye Stability and Analysis of FRET Interaction on DNA Microarrays

Marcel von der Haar, Christopher Heuer, Martin Pähler, Patrick Lindner, Thomas Scheper
and Frank Stahl

**Table S1.** Sets of Genes for which oligos where designed as used in the 96 gene, two-array experiment and the 24 gene, three-array experiment. Selected oligos are marked with an x for each experiment.

| **Gene** | **96 Gene Two-Array Experiment** | **24 Gene Three-Array Experiment** |
| --- | --- | --- |
| 16S pseudouridylate 516 synthase; rsuA | x |  |
| 2-oxoglutarate dehydrogenase  (decarboxylase component); sucA | x | x |
| 2-oxoglutarate dehydrogenase (dihydrolipoyltranssuccinase E2 component); sucB | x |  |
| 30S ribosomal subunit protein S16; rpsP | x |  |
| 30S ribosomal subunit protein S7, initiates assembly; rpsG | x |  |
| 50S ribosomal subunit protein L10; rplJ | x | x |
| 50S ribosomal subunit protein L18; rplR | x |  |
| 50S ribosomal subunit protein L2; rplB | x |  |
| 50S ribosomal subunit protein L23; rplW | x | x |
| 50S ribosomal subunit protein L3; rplC | x |  |
| 50S ribosomal subunit protein L30; rpmD | x |  |
| 50S ribosomal subunit protein L4, regulates expression of S10 operon; rplD | x |  |
| Arabidopsis Control Oligonucleotide | x |  |
| arginine 3rd transport system permease protein; artQ | x | x |
| ATP-binding component of sn-glycerol 3-phosphate transport system; ugpC | x |  |
| bifunctional pyrimidine deaminase/reductase in pathway of riboflavin synthesis; ribD | x |  |
| cell division protein; ftsJ | x |  |
| chaperone Hsp70; DNA biosynthesis; autoregulated heat shock proteins; dnaK | x |  |
| chaperone Hsp70; DNA biosynthesis; autoregulated heat shock proteins; dnaK | x |  |
| chaperone with DnaK; heat shock protein; dnaJ | x |  |
| coproporphyrinogen III oxidase; hemF | x |  |
| cytochrome d terminal oxidase, polypeptide subunit I; cydA | x |  |
| cytochrome o ubiquinol oxidase subunit I; cyoB | x |  |
| cytochrome o ubiquinol oxidase subunit II; cyoA | x | x |
| cytochrome o ubiquinol oxidase subunit III; cyoC | x |  |
| delta(2)-isopentenylpyrophosphate tRNA-adenosine transferase; miaA | x |  |
| DNA biosynthesis; DNA primase; dnaG | x |  |
| DNA polymerase III, chi subunit; holC | x | x |
| DNA-binding, ATP-dependent protease La; heat shock  K-protein; lon | x |  |
| D-ribulose-5-phosphate 3-epimerase; rpe | x |  |
| fermentative D-lactate dehydrogenase, NAD-dependent; ldhA | x |  |
| formate dehydrogenase-O, major subunit; fdoG | x |  |
| fumarate reductase, anaerobic, membrane anchor polypeptide; frdC | x |  |
| fumarate reductase, anaerobic, membrane anchor polypeptide; frdD | x |  |
| galactitol-specific enzyme IIA of phosphotransferase system; gatA | x |  |
| GroEL, chaperone Hsp60, peptide-dependent ATPase, heat shock protein; mopA | x |  |
| GroES, 10 Kd chaperone binds to Hsp60 in pres, Mg-ATP, suppressing its ATPase activity; mopB | x | x |
| GTP-binding export factor binds to signal sequence, GTP and RNA; ffh | x | x |
| heat shock protein hslJ; hslJ | x |  |
| heat shock protein hslVU, ATPase subunit, homologous to chaperones; hslU | x |  |
| heat shock protein hslVU, proteasome-related peptidase subunit; hslV | x |  |
| heat shock protein, chaperone, member of Hsp70 protein family; hscA | x |  |
| heat shock protein, integral membrane protein; htpX | x |  |
| heat shock protein; clpB | x |  |
| heat shock protein; ibpA | x |  |
| heat shock protein; ibpB | x |  |
| host factor I for bacteriophage Q beta replication, a growth-related protein; hfq | x |  |
| internal control | x | x |
| IS186 hypothetical protein; yi81_1 | x |  |
| isocitrate dehydrogenase, specific for NADP+; icdA | x |  |
| mechanosensitive channel; mscL | x |  |
| membrane-bound ATP synthase, F1 sector,  alpha-subunit; atpA | x |  |
| membrane-bound ATP synthase, F1 sector,  alpha-subunit; atpA | x |  |
| membrane-bound ATP synthase, F1 sector, beta-subunit; atpD | x | x |
| membrane-bound ATP synthase, F1 sector, delta-subunit; atpH | x |  |
| membrane-bound ATP synthase, F1 sector,  gamma-subunit; atpG | x | x |
| methylglyoxal synthase; mgsA | x | x |
| multiple antibiotic resistance; transcriptional activator of defense systems; marA | x |  |
| N-acetylglucosamine metabolism; nagD | x |  |
| NADH dehydrogenase I chain F; nuoF | x |  |
| NADH dehydrogenase I chain H; nuoH | x |  |
| NADH dehydrogenase I chain J; nuoJ | x |  |
| NADH dehydrogenase I chain L; nuoL | x | x |
| NADH dehydrogenase I chain M; nuoM | x |  |
| nitrate/nitrite response regulator (sensor NarQ); narP | x |  |
| orf, hypothetical protein; b1541 | x |  |
| orf, hypothetical protein; b1824 | x |  |
| orf, hypothetical protein; b3000 | x | x |
| orf, hypothetical protein; ybgF | x |  |
| orf, hypothetical protein; yccV | x | x |
| orf, hypothetical protein; yfjA | x |  |
| orf, hypothetical protein; yhaL | x | x |
| orf, hypothetical protein; yhfY | x |  |
| outer membrane porin protein; locus of qsr prophage; nmpC | x | x |
| periplasmic protein involved in the tonb-independent uptake of group A colicins; tolB | x | x |
| phage lambda replication; host DNA synthesis; heat shock protein; protein repair; grpE | x |  |
| phosphoenolpyruvate carboxykinase; pckA | x |  |
| probable third cytochrome oxidase, subunit I; appC | x | x |
| PTS enzyme IIAB, mannose-specific; manX | x |  |
| PTS system, N-acetylglucosamine-specific enzyme IIABC; nagE | x |  |
| putative amino acid/amine transport protein; yeaN | x | x |
| putative ATP-binding component of a transport system; ybjZ | x |  |
| putative ATP-binding protein in pho regulon; ybeZ | x |  |
| putative EC 2,1 enzymes; ycjX | x |  |
| putative ligase; yjfG | x | x |
| putative oxidoreductase; ydfI | x |  |
| putative phosphatase; yrfG | x |  |
| putative tagatose 6-phosphate kinase 1; gatZ | x |  |
| putative transport ATPase; yhiD | x | x |
| putative transport system permease protein; yhfT | x |  |
| pyruvate dehydrogenase (decarboxylase component); aceE | x |  |
| serine hydroxymethyltransferase; glyA | x |  |
| sodium-calcium/proton antiporter; chaA | x | x |
| superoxide dismutase, iron; sodB | x |  |
| tagatose-bisphosphate aldolase 1; gatY | x | x |
| UDP-D-galactose:(glucosyl)lipopolysaccharide-1,6- D-galactosyltransferase; rfaB | x |  |

**Table S2.** Influence of presence absence of protective measures on overall spot intensity deviations for Array1 (unprotected vs. 1 mM PBS). ***SS***: Sum of Squares, ***df***: degrees of freedom, ***MS***: Mean of Square Sums, ***F***: F-value, ***p***: p-value corresponding to *F*, ***F_crit_***: critical *F* corresponding to chosen confidence interval (*α* = 0.05)

| **Cy3 Single Dye** | | | | | | |
| --- | --- | --- | --- | --- | --- | --- |
|  | *groups* | *n* | *sum* | *mean* | *variance* |  |
|  | no protection | 10 | 7.68 x 10^4^ | 7.68 x 10^3^ | 4.42 x 10^7^ |  |
|  | 1 mM PBS | 15 | 1.43 x 10^5^ | 9.58 x 10^3^ | 105 x 10^8^ |  |
| *source of deviation* | *SS* | *df* | *MS* | *F* | *p* | *F_crit_* |
| between groups | 2.16 × 10^7^ | 1 | 2.16 x 10^7^ | 0.27 | 0.61 | 4.28 |
| within groups | 1.87 × 10^9^ | 23 | 8.14 x 10^7^ |  |  |  |
| total | 1.89 × 10^9^ | 24 |  |  |  |  |
| **Cy5 Single Dye** | | | | | | |
|  | *groups* | *n* | *sum* | *mean* | *variance* |  |
|  | no protection | 10 | 7.58 x 10^4^ | 7.58 x 10^3^ | 1.10 x 10^8^ |  |
|  | 1 mM PBS | 15 | 9.55 x 10^4^ | 6.37 x 10^3^ | 6.68 x 10^7^ |  |
| *source of deviation* | *SS* | *df* | *MS* | *F* | *p* | *F_crit_* |
| between groups | 8.81 × 10^6^ | 1 | 8.80 x 10^6^ | 0.11 | 0.75 | 4.28 |
| within groups | 1.92 × 10^9^ | 23 | 8.36 x 10^7^ |  |  |  |
| total | 1.93 × 10^9^ | 24 |  |  |  |  |
| **Cy3 Two Dye** | | | | | | |
|  | *groups* | *n* | *sum* | *mean* | *variance* |  |
|  | no protection | 10 | 8.50 × 10^4^ | 8.50 × 10^3^ | 2.68 × 10^7^ |  |
|  | 1 mM PBS | 15 | 2.04 × 10^5^ | 1.36 × 10^4^ | 1.87 × 10^8^ |  |
| *source of deviation* | *SS* | *df* | *MS* | *F* | *p* | *F_crit_* |
| between groups | 1.55 × 10^8^ | 1 | 1.55 × 10^8^ | 1.25 | 0.28 | 4.28 |
| within groups | 2.86 × 10^9^ | 23 | 1.25 × 10^8^ |  |  |  |
| total | 3.02 × 10^9^ | 24 |  |  |  |  |
| **Cy5 Two Dye** | | | | | | |
|  | *groups* | *n* | *sum* | *mean* | *variance* |  |
|  | no protection | 10 | 2.68 × 10^4^ | 2.68 × 10^3^ | 7.15 × 10^6^ |  |
|  | 1 mM PBS | 15 | 8.08 × 10^2^ | 5.39 × 10^3^ | 4.69 × 10^7^ |  |
| *source of deviation* | *SS* | *df* | *MS* | *F* | *p* | *F_crit_* |
| between groups | 4.42 × 10^7^ | 1 | 4.42 × 10^7^ | 1.41 | 0.25 | 4.28 |
| within groups | 7.21 × 10^8^ | 23 | 3.14 × 10^7^ |  |  |  |
| total | 7.66 × 10^8^ | 24 |  |  |  |  |

**Table S3.** Influence of presence absence of protective measures on overall spot intensity deviations for Array2 (unprotected vs. 10 mM ROXS in 1 mM PBS). ***SS***: Sum of Squares, ***df***: degrees of freedom, ***MS***: Mean of Square Sums, ***F***: F-value, ***p***: p-value corresponding to *F*, ***F_crit_***: critical *F* corresponding to chosen confidence interval (*α* = 0.05)

| **Cy3 Single Dye** | | | | | | |
| --- | --- | --- | --- | --- | --- | --- |
|  | *groups* | *n* | *sum* | *mean* | *variance* |  |
|  | no protection | 13 | 1.94 × 10^5^ | 1.49 × 10^4^ | 3.55 × 10^8^ |  |
|  | 1 mM PBS | 14 | 1.24 × 10^5^ | 8.83 × 10^3^ | 1.25 × 10^8^ |  |
| *source of deviation* | *SS* | *df* | *MS* | *F* | *p* | *F_crit_* |
| between groups | 2.49 × 10^8^ | 1 | 2.49 × 10^8^ | 1.06 | 0.31 | 4.24 |
| within groups | 5.89 × 10^9^ | 25 | 2.35 × 10^8^ |  |  |  |
| total | 6.14 × 10^9^ | 26 |  |  |  |  |
| **Cy5 Single Dye** | | | | | | |
|  | *groups* | *n* | *sum* | *mean* | *variance* |  |
|  | no protection | 13 | 1.03 × 10^5^ | 7.90 × 10^3^ | 6.75 × 10^7^ |  |
|  | 1 mM PBS | 14 | 5.28 × 10^4^ | 3.77 × 10^3^ | 9.92 × 10^6^ |  |
| *source of deviation* | *SS* | *df* | *MS* | *F* | *p* | *F_crit_* |
| between groups | 1.15 × 10^8^ | 1 | 1.15 × 10^8^ | 3.05 | 0.09 | 4.24 |
| within groups | 9.39 × 10^8^ | 25 | 3.76 × 10^7^ |  |  |  |
| total | 1.05 × 10^9^ | 26 |  |  |  |  |
| **Cy3 Two Dye** | | | | | | |
|  | *groups* | *n* | *sum* | *mean* | *variance* |  |
|  | no protection | 13 | 1.96 × 10^5^ | 1.51 × 10^4^ | 3.79 × 10^8^ |  |
|  | 1 mM PBS | 14 | 1.25 × 10^5^ | 8.96 × 10^3^ | 7.87 × 10^7^ |  |
| *source of deviation* | *SS* | *df* | *MS* | *F* | *p* | *F_crit_* |
| between groups | 2.54 × 10^8^ | 1 | 2.54 × 10^8^ | 1.14 | 0.30 | 4.24 |
| within groups | 5.57 × 10^9^ | 25 | 2.23 × 10^8^ |  |  |  |
| total | 5.82 × 10^9^ | 26 |  |  |  |  |
| **Cy5 Two Dye** | | | | | | |
|  | *groups* | *n* | *sum* | *mean* | *variance* |  |
|  | no protection | 13 | 5.87 × 10^4^ | 4.51 × 10^3^ | 3.33 × 10^7^ |  |
|  | 1 mM PBS | 14 | 7.03 × 10^4^ | 5.02 × 10^3^ | 2.69 × 10^7^ |  |
| *source of deviation* | *SS* | *df* | *MS* | *F* | *p* | *F_crit_* |
| between groups | 1.75 × 10^6^ | 1 | 1.75 × 10^6^ | 0.06 | 0.81 | 4.24 |
| within groups | 7.48 × 10^8^ | 25 | 2.99 × 10^7^ |  |  |  |
| total | 7.50 × 10^8^ | 26 |  |  |  |  |

**Table S4.** Influence of presence absence of protective measures on overall spot intensity deviations for Array3 (unprotected vs. 50 mM ROXS in 1 mM PBS).

| **Cy3 Single Dye** | | | | | | |
| --- | --- | --- | --- | --- | --- | --- |
|  | *groups* | *n* | *sum* | *mean* | *variance* |  |
|  | no protection | 10 | 9.48 × 10^4^ | 9.48 × 10^3^ | 8.31 × 10^7^ |  |
|  | 1 mM PBS | 14 | 2.06 × 10^5^ | 1.47 × 10^4^ | 1.69 × 10^8^ |  |
| *source of deviation* | *SS* | *df* | *MS* | *F* | *p* | *F_crit_* |
| between groups | 1.61 × 10^8^ | 1 | 1.61 × 10^8^ | 1.20 | 0.29 | 4.30 |
| within groups | 2.95 × 10^9^ | 22 | 1.33 × 10^8^ |  |  |  |
| total | 3.11 × 10^9^ | 23 |  |  |  |  |
| **Cy5 Single Dye** | | | | | | |
|  | *groups* | *n* | *sum* | *mean* | *variance* |  |
|  | no protection | 10 | 1.18 × 10^5^ | 1.18 × 10^4^ | 2.65 × 10^8^ |  |
|  | 1 mM PBS | 14 | 9.29 × 10^4^ | 6.63 × 10^3^ | 7.91 × 10^7^ |  |
| *source of deviation* | *SS* | *df* | *MS* | *F* | *P* | *F_crit_* |
| between groups | 1.55 × 10^8^ | 1 | 1.55 × 10^8^ | 1.00 | 0.33 | 4.30 |
| within groups | 3.41 × 10^9^ | 22 | 1.55 × 10^8^ |  |  |  |
| total | 3.56 × 10^9^ | 23 |  |  |  |  |
| **Cy3 Two Dye** | | | | | | |
|  | *groups* | *n* | *sum* | *mean* | *variance* |  |
|  | no protection | 10 | 6.46 × 10^4^ | 6.46 × 10^3^ | 7.57 × 10^7^ |  |
|  | 1 mM PBS | 14 | 1.12 × 10^5^ | 7.97 × 10^3^ | 1.16 × 10^8^ |  |
| *source of deviation* | *SS* | *Df* | *MS* | *F* | *P* | *F_crit_* |
| between groups | 1.33 × 10^7^ | 1 | 1.33 × 10^7^ | 0.13 | 0.72 | 4.30 |
| within groups | 2.19 × 10^9^ | 22 | 9.97 × 10^7^ |  |  |  |
| total | 2.21 × 10^9^ | 23 |  |  |  |  |
| **Cy5 Two Dye** | | | | | | |
|  | *groups* | *n* | *sum* | *mean* | *variance* |  |
|  | no protection | 10 | 7.39 × 10^4^ | 7.39 × 10^3^ | 5.42 × 10^7^ |  |
|  | 1 mM PBS | 14 | 2.17 × 10^5^ | 1.55 × 10^4^ | 2.19 × 10^8^ |  |
| *source of deviation* | *SS* | *df* | *MS* | *F* | *p* | *F_crit_* |
| between groups | 3.85 × 10^8^ | 1 | 3.85 × 10^8^ | 2.54 | 0.12 | 4.30 |
| within groups | 3.33 × 10^9^ | 22 | 1.51 × 10^8^ |  |  |  |
| total | 3.72 × 10^9^ | 23 |  |  |  |  |


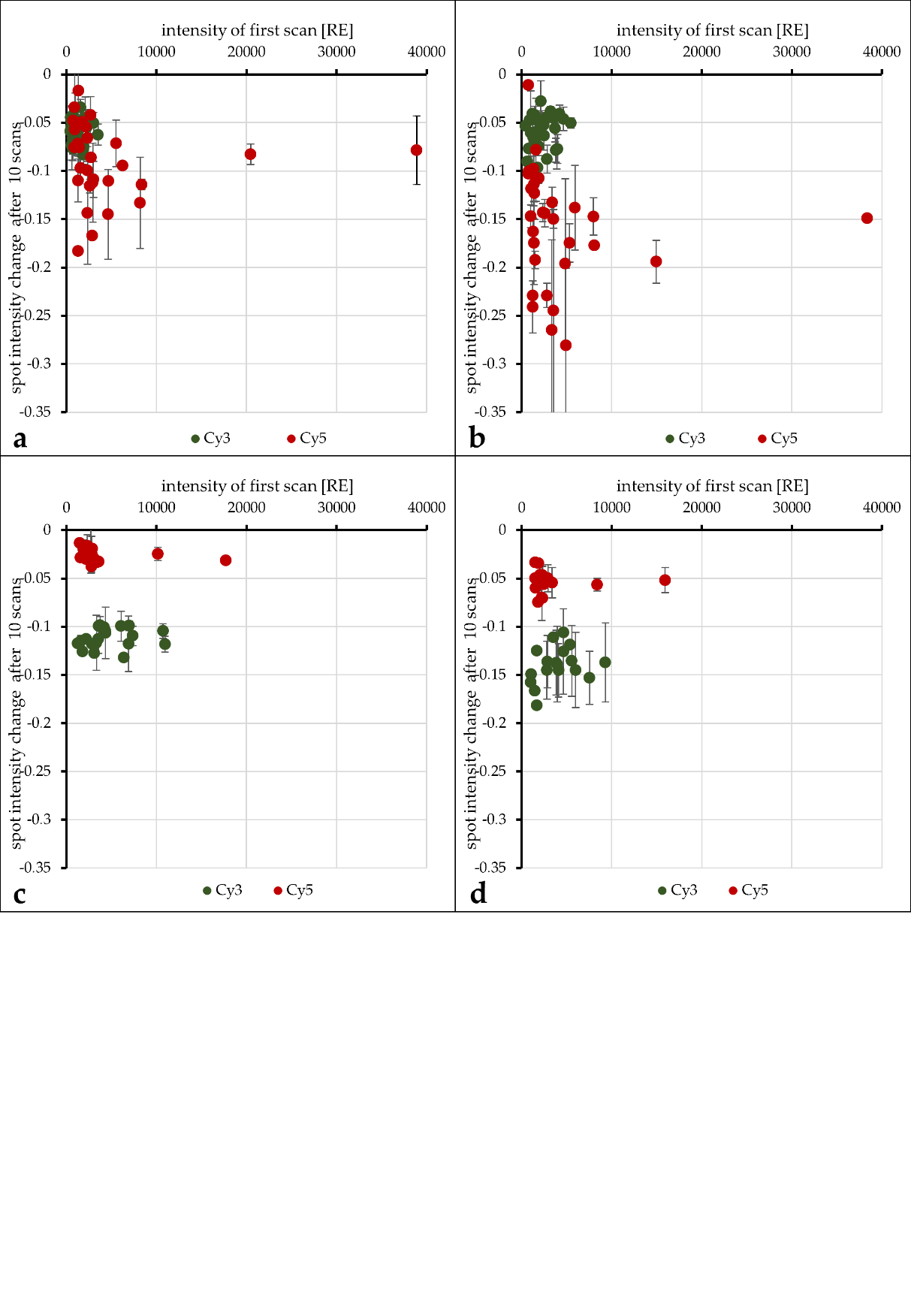


**Figure S1.** Influence of spot intensity level on spot intensity percent change after 10 scans. (**a**) unprotected single dye spots; (**b**) unprotected two dye spots; (**c**) 1 mM ROXS in 1 mM PBS protected single dye spots; (**d**) 1 mM ROXS in 1 mM PBS protected two dye spots. Error indicators are simple standard deviations.

© 2016 by the authors; licensee MDPI, Basel, Switzerland. This article is an open access article distributed under the terms and conditions of the Creative Commons by Attribution (CC-BY) license (http://creativecommons.org/licenses/by/4.0/).
