# Supplementary material for: Optimization of Cyanine Dye Stability and Analysis of FRET Interaction on DNA Microarrays
Source: Biology (Basel). 2016 Nov 30;5(4):47. doi: 10.3390/biology5040047 (PMC5192427; doi:10.3390/biology5040047)
Supplement: Supplementary file 2 [file biology-05-00047-s002.pdf]

Supplementary Materials: Optimization of Cyanine Dye Stability and Analysis of FRET Interaction on DNA Microarrays

Marcel von der Haar, Christopher Heuer, Martin Pähler, Patrick Lindner, Thomas Scheper and Frank Stahl

Table S1. Sets of Genes for which oligos where designed as used in the 96 gene, two-array experiment and the 24 gene, three-array experiment. Selected oligos are marked with an x for each experiment.

Gene	96 Gene Two-Array Experiment	24 Gene Three-Array Experiment
16S pseudouridylate 516 synthase; rsuA	x	
2-oxoglutarate dehydrogenase (decarboxylase component); sucA	x	x
2-oxoglutarate dehydrogenase (dihydrolipoyltranssuccinase E2 component); sucB	x	
30S ribosomal subunit protein S16; rpsP	x	
30S ribosomal subunit protein S7, initiates assembly; rpsG	x	
50S ribosomal subunit protein L10; rplJ	x	x
50S ribosomal subunit protein L18; rplR	x	
50S ribosomal subunit protein L2; rplB	x	
50S ribosomal subunit protein L23; rplW	x	x
50S ribosomal subunit protein L3; rplC	x	
50S ribosomal subunit protein L30; rpmD	x	
50S ribosomal subunit protein L4, regulates expression of S10 operon; rplD	x	
Arabidopsis Control Oligonucleotide	x	
arginine 3rd transport system permease protein; artQ	x	x
ATP-binding component of sn-glycerol 3-phosphate transport system; ugpC	x	
bifunctional pyrimidine deaminase/reductase in pathway of riboflavin synthesis; ribD	x	
cell division protein; ftsJ	x	
chaperone Hsp70; DNA biosynthesis; autoregulated heat shock proteins; dnaK	x	
chaperone Hsp70; DNA biosynthesis; autoregulated heat shock proteins; dnaK	x	
chaperone with DnaK; heat shock protein; dnaJ	x	
coproporphyrinogen III oxidase; hemF	x	
cytochrome d terminal oxidase, polypeptide subunit I; cydA	x	
cytochrome o ubiquinol oxidase subunit I; cyoB	x	
cytochrome o ubiquinol oxidase subunit II; cyoA	x	x
cytochrome o ubiquinol oxidase subunit III; cyoC	x	
delta(2)-isopentenylpyrophosphate tRNA-adenosine transferase; miaA	x	
DNA biosynthesis; DNA primase; dnaG	x	
DNA polymerase III, chi subunit; holC	x	x

DNA-binding, ATP-dependent protease La; heat shock		
K-protein; lon	x	
D-ribulose-5-phosphate 3-epimerase; rpe	x	
fermentative D-lactate dehydrogenase, NAD-dependent; ldhA	x	
formate dehydrogenase-O, major subunit; fdoG	x	
fumarate reductase, anaerobic, membrane anchor polypeptide; frdC	x	
fumarate reductase, anaerobic, membrane anchor polypeptide; frdD	x	
galactitol-specific enzyme IIA of phosphotransferase system; gatA	x	
GroEL, chaperone Hsp60, peptide-dependent ATPase, heat shock protein; mopA	x	
GroES, 10 Kd chaperone binds to Hsp60 in pres, Mg-ATP, suppressing its ATPase activity; mopB	x	x
GTP-binding export factor binds to signal sequence, GTP and RNA; ffh	x	x
heat shock protein hslJ; hslJ	x	
heat shock protein hslVU, ATPase subunit, homologous to chaperones; hslU	x	
heat shock protein hslVU, proteasome-related peptidase subunit; hslV	x	
heat shock protein, chaperone, member of Hsp70 protein family; hscA	x	
heat shock protein, integral membrane protein; htpX	x	
heat shock protein; clpB	x	
heat shock protein; ibpA	x	
heat shock protein; ibpB	x	
host factor I for bacteriophage Q beta replication, a growth-related protein; hfq	x	
internal control	x	x
IS186 hypothetical protein; yi81_1	x	
isocitrate dehydrogenase, specific for NADP+; icdA	x	
mechanosensitive channel; mscL	x	
membrane-bound ATP synthase, F1 sector, alpha-subunit; atpA	x	
membrane-bound ATP synthase, F1 sector, alpha-subunit; atpA	x	
membrane-bound ATP synthase, F1 sector, beta-subunit; atpD	x	x
membrane-bound ATP synthase, F1 sector, delta-subunit; atpH	x	
membrane-bound ATP synthase, F1 sector, gamma-subunit; atpG	x	x
methylglyoxal synthase; mgsA	x	x
multiple antibiotic resistance; transcriptional activator of defense systems; marA	x	
N-acetylglucosamine metabolism; nagD	x	
NADH dehydrogenase I chain F; nuoF	x	
NADH dehydrogenase I chain H; nuoH	x	

NADH dehydrogenase I chain J; nuoJ	x	
NADH dehydrogenase I chain L; nuoL	x	x
NADH dehydrogenase I chain M; nuoM	x	
nitrate/nitrite response regulator (sensor NarQ); narP	x	
orf, hypothetical protein; b1541	x	
orf, hypothetical protein; b1824	x	
orf, hypothetical protein; b3000	x	x
orf, hypothetical protein; ybgF	x	
orf, hypothetical protein; yccV	x	x
orf, hypothetical protein; yfjA	x	
orf, hypothetical protein; yhaL	x	x
orf, hypothetical protein; yhfY	x	
outer membrane porin protein; locus of qsr prophage; nmpC	x	x
periplasmic protein involved in the tonb-independent uptake of group A colicins; tolB	x	x
phage lambda replication; host DNA synthesis; heat shock protein; protein repair; grpE	x	
phosphoenolpyruvate carboxykinase; pckA	x	
probable third cytochrome oxidase, subunit I; appC	x	x
PTS enzyme IIAB, mannose-specific; manX	x	
PTS system, N-acetylglucosamine-specific enzyme IIABC; nagE	x	
putative amino acid/amine transport protein; yeaN	x	x
putative ATP-binding component of a transport system; ybjZ	x	
putative ATP-binding protein in pho regulon; ybeZ	x	
putative EC 2,1 enzymes; ycjX	x	
putative ligase; yjfG	x	x
putative oxidoreductase; ydfI	x	
putative phosphatase; yrfG	x	
putative tagatose 6-phosphate kinase 1; gatZ	x	
putative transport ATPase; yhiD	x	x
putative transport system permease protein; yhfT	x	
pyruvate dehydrogenase (decarboxylase component); aceE	x	
serine hydroxymethyltransferase; glyA	x	
sodium-calcium/proton antiporter; chaA	x	x
superoxide dismutase, iron; sodB	x	
tagatose-bisphosphate aldolase 1; gatY	x	x
UDP-D-galactose:(glucosyl)lipopolysaccharide-1,6- D-galactosyltransferase; rfaB	x	

Table S2. Influence of presence absence of protective measures on overall spot intensity deviations for Array1 (unprotected vs. 1 mM PBS). ***SS***: Sum of Squares, ***df***: degrees of freedom, ***MS***: Mean of Square Sums, ***F***: F-value, ***p***: p-value corresponding to *F*, $\boldsymbol{F_{crit}}$: critical *F* corresponding to chosen confidence interval (α = 0.05)

			**Cy3 Single Dye**			
	groups	*n*	*sum*	*mean*	*variance*	
	no protection	10	7.68×10^4	7.68×10^3	4.42×10^7	
	1 mM PBS	15	1.43×10^5	9.58×10^3	105×10^8	
source of deviation	*SS*	*df*	*MS*	*F*	*p*	F_{crit}
between groups	2.16×10^7	1	2.16×10^7	0.27	0.61	4.28
within groups	1.87×10^9	23	8.14×10^7			
total	1.89×10^9	24				
			**Cy5 Single Dye**			
	groups	*n*	*sum*	*mean*	*variance*	
	no protection	10	7.58×10^4	7.58×10^3	1.10×10^8	
	1 mM PBS	15	9.55×10^4	6.37×10^3	6.68×10^7	
source of deviation	*SS*	*df*	*MS*	*F*	*p*	F_{crit}
between groups	8.81×10^6	1	8.80×10^6	0.11	0.75	4.28
within groups	1.92×10^9	23	8.36×10^7			
total	1.93×10^9	24				
			**Cy3 Two Dye**			
	groups	*n*	*sum*	*mean*	*variance*	
	no protection	10	8.50×10^4	8.50×10^3	2.68×10^7	
	1 mM PBS	15	2.04×10^5	1.36×10^4	1.87×10^8	
source of deviation	*SS*	*df*	*MS*	*F*	*p*	F_{crit}
between groups	1.55×10^8	1	1.55×10^8	1.25	0.28	4.28
within groups	2.86×10^9	23	1.25×10^8			
total	3.02×10^9	24				
			**Cy5 Two Dye**			
	groups	*n*	*sum*	*mean*	*variance*	
	no protection	10	2.68×10^4	2.68×10^3	7.15×10^6	
	1 mM PBS	15	8.08×10^2	5.39×10^3	4.69×10^7	
source of deviation	*SS*	*df*	*MS*	*F*	*p*	F_{crit}
between groups	4.42×10^7	1	4.42×10^7	1.41	0.25	4.28
within groups	7.21×10^8	23	3.14×10^7			
total	7.66×10^8	24				

Table S3. Influence of presence absence of protective measures on overall spot intensity deviations for Array2 (unprotected vs. 10 mM ROXS in 1 mM PBS). ***SS***: Sum of Squares, ***df***: degrees of freedom, ***MS***: Mean of Square Sums, ***F***: F-value, ***p***: p-value corresponding to *F*, $\boldsymbol{F_{crit}}$: critical *F* corresponding to chosen confidence interval (α = 0.05)

**Cy3 Single Dye**						
	groups	*n*	*sum*	*mean*	*variance*	
	no protection	13	1.94×10^5	1.49×10^4	3.55×10^8	
	1 mM PBS	14	1.24×10^5	8.83×10^3	1.25×10^8	
source of deviation	*SS*	*df*	*MS*	*F*	*p*	F_{crit}
between groups	2.49×10^8	1	2.49×10^8	1.06	0.31	4.24
within groups	5.89×10^9	25	2.35×10^8			
total	6.14×10^9	26				
**Cy5 Single Dye**						
	groups	*n*	*sum*	*mean*	*variance*	
	no protection	13	1.03×10^5	7.90×10^3	6.75×10^7	
	1 mM PBS	14	5.28×10^4	3.77×10^3	9.92×10^6	
source of deviation	*SS*	*df*	*MS*	*F*	*p*	F_{crit}
between groups	1.15×10^8	1	1.15×10^8	3.05	0.09	4.24
within groups	9.39×10^8	25	3.76×10^7			
total	1.05×10^9	26				
**Cy3 Two Dye**						
	groups	*n*	*sum*	*mean*	*variance*	
	no protection	13	1.96×10^5	1.51×10^4	3.79×10^8	
	1 mM PBS	14	1.25×10^5	8.96×10^3	7.87×10^7	
source of deviation	*SS*	*df*	*MS*	*F*	*p*	F_{crit}
between groups	2.54×10^8	1	2.54×10^8	1.14	0.30	4.24
within groups	5.57×10^9	25	2.23×10^8			
total	5.82×10^9	26				
**Cy5 Two Dye**						
	groups	*n*	*sum*	*mean*	*variance*	
	no protection	13	5.87×10^4	4.51×10^3	3.33×10^7	
	1 mM PBS	14	7.03×10^4	5.02×10^3	2.69×10^7	
source of deviation	*SS*	*df*	*MS*	*F*	*p*	F_{crit}
between groups	1.75×10^6	1	1.75×10^6	0.06	0.81	4.24
within groups	7.48×10^8	25	2.99×10^7			
total	7.50×10^8	26				

Table S4. Influence of presence absence of protective measures on overall spot intensity deviations for Array3 (unprotected vs. 50 mM ROXS in 1 mM PBS).

**Cy3 Single Dye**						
	groups	*n*	*sum*	*mean*	*variance*	
	no protection	10	9.48×10^4	9.48×10^3	8.31×10^7	
	1 mM PBS	14	2.06×10^5	1.47×10^4	1.69×10^8	
source of deviation	*SS*	*df*	*MS*	*F*	*p*	F_{crit}
between groups	1.61×10^8	1	1.61×10^8	1.20	0.29	4.30
within groups	2.95×10^9	22	1.33×10^8			
total	3.11×10^9	23				
**Cy5 Single Dye**						
	groups	*n*	*sum*	*mean*	*variance*	
	no protection	10	1.18×10^5	1.18×10^4	2.65×10^8	
	1 mM PBS	14	9.29×10^4	6.63×10^3	7.91×10^7	
source of deviation	*SS*	*df*	*MS*	*F*	*P*	F_{crit}
between groups	1.55×10^8	1	1.55×10^8	1.00	0.33	4.30
within groups	3.41×10^9	22	1.55×10^8			
total	3.56×10^9	23				
**Cy3 Two Dye**						
	groups	*n*	*sum*	*mean*	*variance*	
	no protection	10	6.46×10^4	6.46×10^3	7.57×10^7	
	1 mM PBS	14	1.12×10^5	7.97×10^3	1.16×10^8	
source of deviation	*SS*	*Df*	*MS*	*F*	*P*	F_{crit}
between groups	1.33×10^7	1	1.33×10^7	0.13	0.72	4.30
within groups	2.19×10^9	22	9.97×10^7			
total	2.21×10^9	23				
**Cy5 Two Dye**						
	groups	*n*	*sum*	*mean*	*variance*	
	no protection	10	7.39×10^4	7.39×10^3	5.42×10^7	
	1 mM PBS	14	2.17×10^5	1.55×10^4	2.19×10^8	
source of deviation	*SS*	*df*	*MS*	*F*	*p*	F_{crit}
between groups	3.85×10^8	1	3.85×10^8	2.54	0.12	4.30
within groups	3.33×10^9	22	1.51×10^8			
total	3.72×10^9	23				

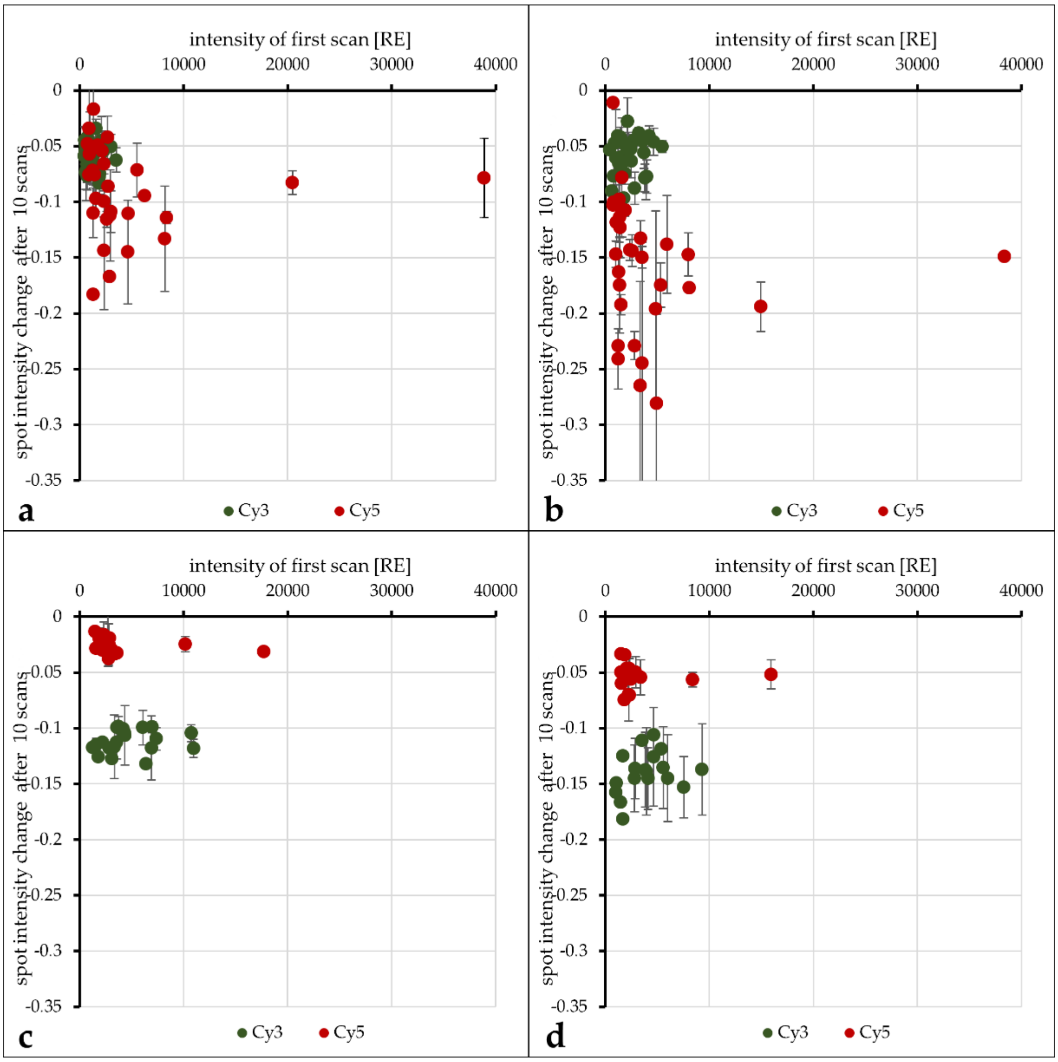

Figure S1. Influence of spot intensity level on spot intensity percent change after 10 scans. (**a**) unprotected single dye spots; (**b**) unprotected two dye spots; (**c**) 1 mM ROXS in 1 mM PBS protected single dye spots; (**d**) 1 mM ROXS in 1 mM PBS protected two dye spots. Error indicators are simple standard deviations.

© 2016 by the authors; licensee MDPI, Basel, Switzerland. This article is an open access article distributed under the terms and conditions of the Creative Commons by Attribution (CC-BY) license (http://creativecommons.org/licenses/by/4.0/).
